# High sugar-sweetened beverage intake predicts adverse physical, emotional, and sleep health trajectories in adolescents: a 4-year prospective cohort study

**DOI:** 10.3389/fpubh.2025.1754072

**Published:** 2026-01-14

**Authors:** Zeyu Zhang, Wanjun Li, Min Zhang, Yike Zhang

**Affiliations:** 1 School of Educational Science, Xinjiang Normal University, Urumqi, China; 2 Biological Education Teaching and Research Group, Chongqing Tongnan Experimental Middle School, Chongqing, China; 3 English Education Teaching and Research Group, Suqian Chongwen Junior Middle School, Suqian, China; 4 School of Accounting, Nanjing University of Finance and Economics, Nanjing, Jiangsu, China

**Keywords:** adolescent health, cardiometabolic risk, depressive symptoms, longitudinal cohort, public health, sleep quality, sugar-sweetened beverages

## Abstract

**Background:**

Excessive consumption of sugar-sweetened beverages (SSBs) is a major source of added sugars among adolescents and has been associated with adverse cardiometabolic, emotional, and sleep-related outcomes. However, longitudinal evidence from Asian populations remains limited. This 4-year prospective cohort study examined the associations between SSB intake and multiple health trajectories during adolescence.

**Methods:**

A total of 1,204 adolescents (baseline age: 12.41 ± 0.89 years; 51.2% girls) from 12 secondary schools in Chongqing, China, were followed annually from 2021 to 2024. SSB intake was assessed using a validated food frequency questionnaire adapted for use among Chinese adolescents. Outcomes included body mass index (BMI), body fat percentage, systolic blood pressure, depressive symptoms (CES-D), academic burnout, and sleep quality (Pittsburgh Sleep Quality Index, PSQI). Longitudinal associations were examined using linear mixed-effects models adjusted for age, sex, socioeconomic status, physical activity, screen time, and baseline BMI.

**Results:**

Higher SSB intake was associated with significantly steeper increases in BMI (mean increase: +2.87 vs. +1.90 kg/m^2^), body fat percentage (+4.6% vs. +2.1%), systolic blood pressure (+4.8 vs. +2.1 mmHg), depressive symptoms (CES-D: +6.0 vs. +2.0 points), and sleep disturbance (PSQI: +1.3 vs. +0.4 points) compared with the lowest intake tertile (all *p* < 0.01). Adolescents in the highest SSB tertile exhibited a 62% greater increase in depressive symptoms over 4 years. Participants who reduced SSB intake by ≥30% showed significantly smaller increases across physical, emotional, and sleep-related outcomes.

**Conclusion:**

High SSB intake is prospectively associated with unfavorable cardiometabolic, emotional, and sleep-related health trajectories during adolescence. Reducing SSB consumption represents a promising and actionable strategy for school-based health promotion and chronic disease prevention among Asian youth.

## Introduction

Excessive consumption of sugar-sweetened beverages (SSBs) among adolescents has emerged as a major global public health concern. Population-based surveillance consistently shows that adolescents frequently exceed recommended limits for added sugar intake, with SSBs constituting a dominant source of dietary sugars (1, 2). A substantial body of epidemiological evidence has linked high SSB consumption to increased risks of obesity, insulin resistance, hypertension, systemic inflammation, and other cardiometabolic disorders during adolescence and early adulthood (3).

Beyond cardiometabolic consequences, accumulating evidence indicates that SSB intake is also associated with adverse psychosocial and behavioral outcomes, including emotional dysregulation, sleep disturbances, and academic difficulties (4, 5). Experimental and mechanistic studies suggest that high sugar intake—particularly in liquid form—may disrupt neuroendocrine and circadian regulation through altered cortisol secretion and glycemic fluctuations, thereby affecting sleep–wake rhythms and emotional stability (6). However, much of the existing literature relies on cross-sectional designs, limiting the ability to establish temporal ordering or to distinguish stable between-individual differences from within-individual developmental changes over time.

Importantly, longitudinal evidence from Asian adolescent populations remains limited. Adolescents in East Asia experience distinctive sociocultural contexts, academic pressures, dietary environments, and sleep patterns that may modify the health effects of SSB consumption compared with Western settings (7, 8). Moreover, prior longitudinal studies have predominantly focused on weight-related outcomes, often neglecting emotional and sleep-related domains that are particularly salient during adolescence—a critical period for psychosocial and circadian development (9).

To address these gaps, the present 4-year prospective cohort study investigated longitudinal patterns of SSB intake and their associations with physical, emotional, and sleep-related health outcomes among Chinese adolescents. Specifically, the study aimed to: (1) characterize trajectories of SSB consumption across adolescence; (2) examine prospective associations between SSB intake and changes in adiposity, depressive symptoms, and sleep quality; and (3) generate evidence to inform school-based dietary policies and adolescent health promotion strategies (10, 11).

## Methods

### Study design and participants

This prospective cohort study was conducted from September 2021 to July 2024 in Chongqing Municipality, China. A stratified cluster sampling strategy was used to select 12 public secondary schools representing diverse geographic locations and socioeconomic backgrounds. All students enrolled in selected classes were invited to participate.

Of the 1,320 eligible adolescents, 1,204 completed baseline assessments at Time 1 (T1), yielding a response rate of 91.2%. Follow-up assessments were conducted annually at four time points (T1–T4). Retention rates remained high across waves, with an overall four-year retention of 88.9%. Patterns of attrition were examined and did not differ significantly by sex, baseline SSB intake, or primary health outcomes (*p* > 0.10), supporting the assumption that missing data were missing at random.

### Inclusion criteria

- Age 11–14 years at baseline.
- Enrollment in a participating school with anticipated four-year follow-up.
- Provision of written informed consent from a parent or legal guardian and assent from the adolescent.

### Exclusion criteria

- Diagnosed chronic endocrine or psychiatric disorders.
- Long-term use of medications known to affect body weight, mood, or sleep.
- Self-reported extreme caloric restriction or diagnosed eating disorders.
- Medical conditions that could interfere with repeated measurements.

The study protocol was reviewed and approved by the Ethics Review Committee of the School of Educational Science, Xinjiang Normal University. Prior to participation, all adolescents and their legal guardians were fully informed about the study objectives, procedures, and potential risks, and written informed consent from guardians and assent from adolescents were obtained in accordance with the Declaration of Helsinki.

### Measurements

#### Sugar-sweetened beverage (SSB) intake

SSB intake was assessed at each annual wave using a previously used food frequency questionnaire (FFQ) adapted for use among Chinese adolescents, based on established FFQ instruments for beverage consumption (12). The FFQ captured usual intake frequency and portion size for major categories of sugar-sweetened beverages, including carbonated soft drinks, sweetened tea beverages, juice drinks with added sugar, sports drinks, and energy drinks.

Average daily SSB intake (mL/day) was calculated by multiplying reported consumption frequency by standardized portion sizes and converting weekly intake to daily estimates (13). The FFQ primarily captured liquid sources of added sugars; intake of solid sugary foods was not included and is addressed as a limitation.

Participants were categorized into tertiles based on baseline SSB intake. The approximate cut-points were:

- Low tertile: < ~300 mL/day
- Medium tertile: ~300–500 mL/day
- High tertile: > ~ 500 mL/day

#### Anthropometrics and body composition

Anthropometrics were collected by trained assessors. To minimize inter-rater variability, assessors received unified training annually, followed identical standard operating procedures, and equipment was regularly calibrated. Whenever possible, the same assessment team covered the same schools across waves. Height was measured to the nearest 0.1 cm using a SECA portable stadiometer, and body weight was measured to the nearest 0.1 kg using a calibrated Tanita digital analyzer. Body mass index (BMI) was calculated as weight (kg) divided by height squared (m^2^).

Body fat percentage was assessed using bioelectrical impedance analysis (InBody 770) (14), which has demonstrated acceptable validity and reliability in adolescent populations (15). In sensitivity analyses, BMI z-scores standardized for age and sex were calculated according to World Health Organization (WHO) growth reference standards.

#### Blood pressure

Resting blood pressure was measured twice on the right arm using a calibrated OMRON HBP-1300 electronic sphygmomanometer after a minimum of 5 min of seated rest. The average of the two readings was used for analysis. Measurements were conducted by trained personnel following standardized procedures.

#### Depressive symptoms

Depressive symptoms were assessed annually using the 20-item Center for Epidemiological Studies–Depression Scale (CES-D). Items were rated on a 4-point Likert scale, with higher scores indicating greater depressive symptom severity. In the present sample, internal consistency was good across waves (Cronbach’s *α* = 0.87) (16).

#### Sleep duration and sleep quality

Sleep quality and sleep duration were assessed annually using the Pittsburgh Sleep Quality Index (PSQI) (3), a widely used and validated instrument in adolescent populations. The PSQI global score was used as the primary indicator of sleep quality, with higher scores reflecting poorer sleep. Self-reported average nightly sleep duration (hours/night) was examined in supplementary analyses.

#### Academic burnout

Academic burnout was measured using the Chinese Adolescent Learning Burnout Scale developed by Wu et al. (17), which includes three dimensions: emotional exhaustion, academic alienation, and reduced sense of accomplishment. This variable was included as a supplementary psychosocial indicator. This scale has been widely used in Chinese adolescent populations (18).

#### Covariates

Covariates were selected *a priori* based on established associations with SSB consumption and adolescent health outcomes and included:

- age and sex
- socioeconomic status (parental education and household income)
- physical activity assessed using the International Physical Activity Questionnaire (IPAQ)
- daily screen time
- baseline BMI (or BMI z-score in sensitivity analyses)

### Statistical analysis

Descriptive statistics were calculated for all variables and are presented as means ± standard deviations (SD) for continuous variables and percentages for categorical variables. Missing data across waves were handled using maximum likelihood estimation under the assumption of missing at random.

Longitudinal associations between SSB intake and physical, emotional, and sleep-related outcomes were examined using linear mixed-effects models (LMMs) with random intercepts to account for within-individual correlation across repeated measures. Time (wave), SSB intake group, and their interaction were included as fixed effects. The general model specification was:


$$Y_{\mathrm{it}}=\beta_{0}+\beta_{1}({\mathrm{SSB}}_{i})+\beta_{2}(\text{time})+\beta_{3}({\mathrm{SSB}}_{i}\times {\text{time}}_{t})+u_{i}+\epsilon_{\mathrm{it}}$$

where Y_it_ represents the outcome for individual i at time t, u_i_ is the random intercept capturing stable between-person differences, and ϵ_it_ is the residual error.

Adjusted models additionally included age, sex, socioeconomic status, physical activity, screen time, and baseline BMI (or BMI z-score). Model-estimated marginal means and 95% confidence intervals are reported for longitudinal trajectories.

Sensitivity analyses were conducted by excluding adolescents classified as obese at baseline and by additionally adjusting for total daily caloric intake. Results were consistent across sensitivity analyses.

All statistical tests were two-sided, with statistical significance set at *p* < 0.05. Analyses were performed using SPSS version 26.0 and R version 4.3.0.

## Results

A total of 1,204 adolescents were included in the baseline analytic sample. Table 1 summarizes participants’ baseline characteristics, including demographic variables, sugar-sweetened beverage (SSB) intake, anthropometric indicators, depressive symptoms, and sleep quality. Continuous variables are presented as means ± standard deviations (SD), and categorical variables as percentages.

**Table 1:** Baseline characteristics of the study participants (*N* = 1,204).

Variable	Mean ± SD
Age	12.41 ± 0.89
Girls (%)	51.2
SSB intake (mL/day)	438 ± 186
BMI (kg/m^2^)	19.72 ± 3.41
Body fat (%)	22.8 ± 7.3
CES-D score	11.6 ± 6.1
PSQI score	4.8 ± 2.1

In sensitivity analyses, BMI z-scores standardized for age and sex were additionally calculated based on WHO growth reference data, yielding consistent results (see Supplementary Table S1).

At baseline, the mean age of participants was 12.41 ± 0.89 years, and 51.2% were girls. Average daily SSB intake was 438 ± 186 mL/day. The mean body mass index (BMI) was 19.72 ± 3.41 kg/m^2^, with an average body fat percentage of 22.8 ± 7.3%. Mean scores for depressive symptoms and sleep quality were 11.6 ± 6.1 on the CES-D and 4.8 ± 2.1 on the PSQI, respectively, indicating generally low levels of depressive symptoms and relatively good sleep quality at study entry.

To account for age- and sex-related growth variability during adolescence, BMI z-scores standardized according to the World Health Organization (WHO) growth reference were additionally calculated as a sensitivity analysis. Analyses based on BMI z-scores yielded results highly consistent with those obtained using raw BMI values.

Detailed descriptive statistics and longitudinal estimates for BMI z-scores are presented in Supplementary Table S1, which reports four-year changes in BMI z-scores stratified by baseline SSB intake tertiles. Between-group differences in longitudinal BMI z-score trajectories were examined using linear mixed-effects models with random intercepts, adjusting for age, sex, socioeconomic status, physical activity, screen time, and baseline BMI z-score. The group × time interaction was statistically significant (*p* < 0.01), indicating differential adiposity trajectories across SSB intake levels.

Overall, these findings demonstrate that the association between higher SSB intake and accelerated adiposity gain is robust to age- and sex-standardized anthropometric assessment, supporting the validity of the main analyses based on raw BMI (Table 2).

**Table 2:** Four-year changes in physical health indicators by baseline SSB intake level.

Group	BMI change (kg/m^2^, M ± SD)	Body fat change (%)	SBP change (mmHg)
Low SSB	+1.90 ± 0.84	+2.1 ± 1.6	+2.1 ± 3.2
Medium SSB	+2.40 ± 0.91	+3.3 ± 1.9	+3.4 ± 3.6
High SSB	+2.87 ± 1.02	+4.6 ± 2.2	+4.8 ± 4.1

Findings were consistent using WHO BMI-for-age z-scores (Supplementary Table S1).

### Trajectories of SSB intake

**Figure 1:**
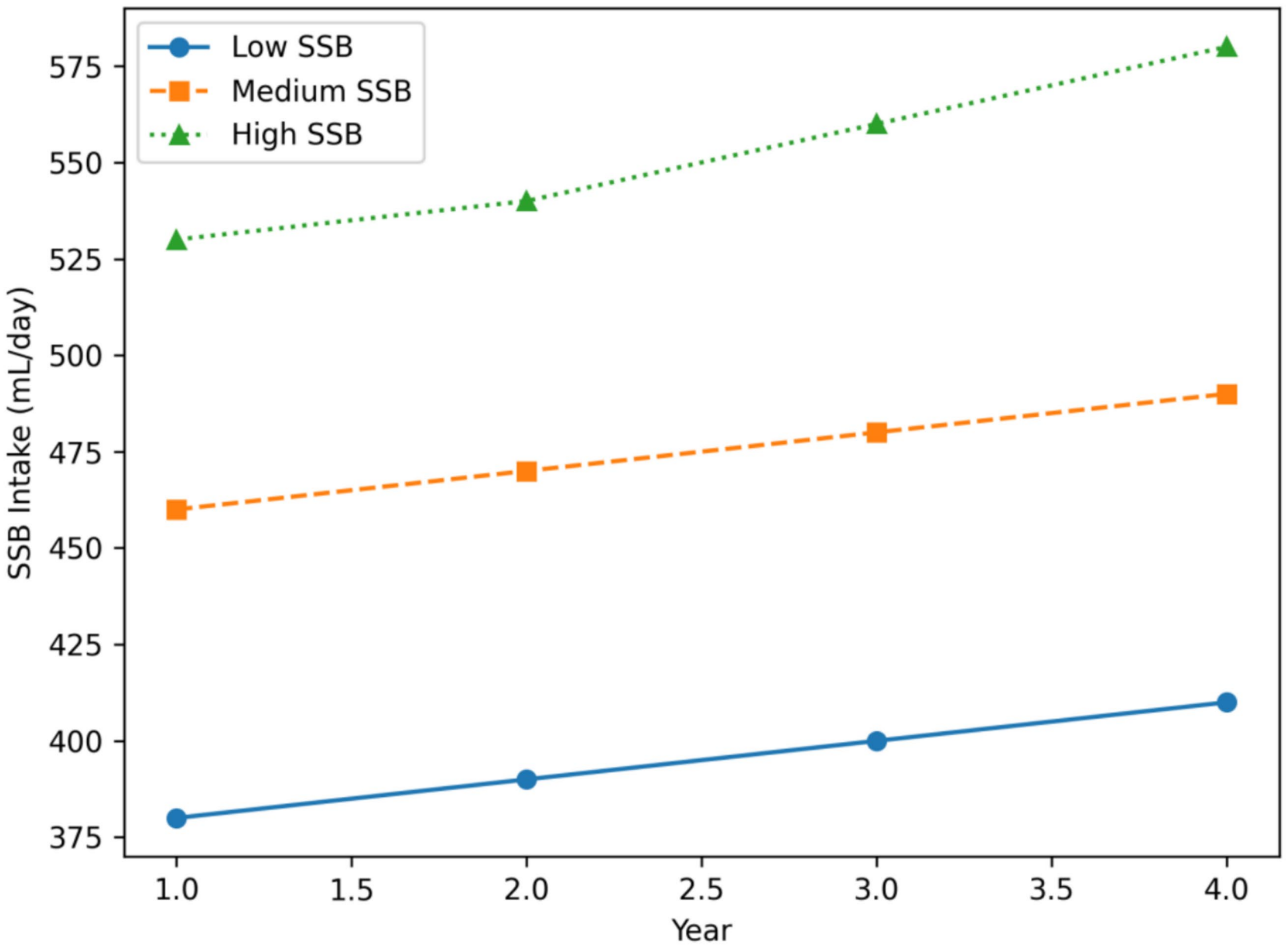
Model-estimated trajectories of sugar-sweetened beverage (SSB) intake across four annual waves (T1–T4) by baseline SSB intake tertile. Lines represent estimated marginal means derived from linear mixed-effects models with random intercepts, and shaded areas indicate 95% confidence intervals. A significant group × time interaction was observed (*p* < 0.001), indicating differential longitudinal changes in SSB intake across tertiles.

As illustrated in Figure 1, adolescents in the high–SSB tertile exhibited a pronounced upward trajectory in sugar-sweetened beverage consumption across the four-year follow-up, with model-estimated intake increasing from approximately 498 mL/day at baseline to 580 mL/day at Year 4. In contrast, adolescents in the low–SSB tertile maintained relatively stable intake levels over time, showing only minor fluctuations across waves (2, 12).

Linear mixed-effects models revealed a significant group × time interaction (*p* < 0.001), indicating that longitudinal changes in SSB intake differed significantly across tertiles. The medium–SSB tertile demonstrated a modest but statistically significant increase in consumption over time (*p* < 0.05), with intake trajectories consistently positioned between those of the low- and high-intake groups. Together, these findings suggest that adolescents with higher baseline SSB consumption are more likely to sustain and further increase intake across adolescence, whereas low consumers show comparatively stable patterns.

### Changes in physical health indicators

Table 2 summarizes four-year changes in physical health indicators, including BMI, body fat percentage, and systolic blood pressure, stratified by baseline SSB intake tertiles. Adolescents in the highest SSB tertile exhibited the largest increase in BMI over follow-up (mean change: +2.87 ± 1.02 kg/m^2^), which was approximately 1 kg/m^2^ greater than the increase observed in the lowest tertile (+1.90 ± 0.84 kg/m^2^). Parallel dose–response patterns were observed for body fat percentage and systolic blood pressure, with progressively steeper increases across low, medium, and high SSB intake groups (12, 14, 19).

Between-group differences in longitudinal changes were examined using linear mixed-effects models adjusting for age, sex, socioeconomic status, physical activity, screen time, and baseline BMI. Significant group × time interaction effects were detected for all three outcomes (all *p* < 0.01), indicating differential trajectories of physical health indicators according to baseline SSB intake level.

**Figure 2:**
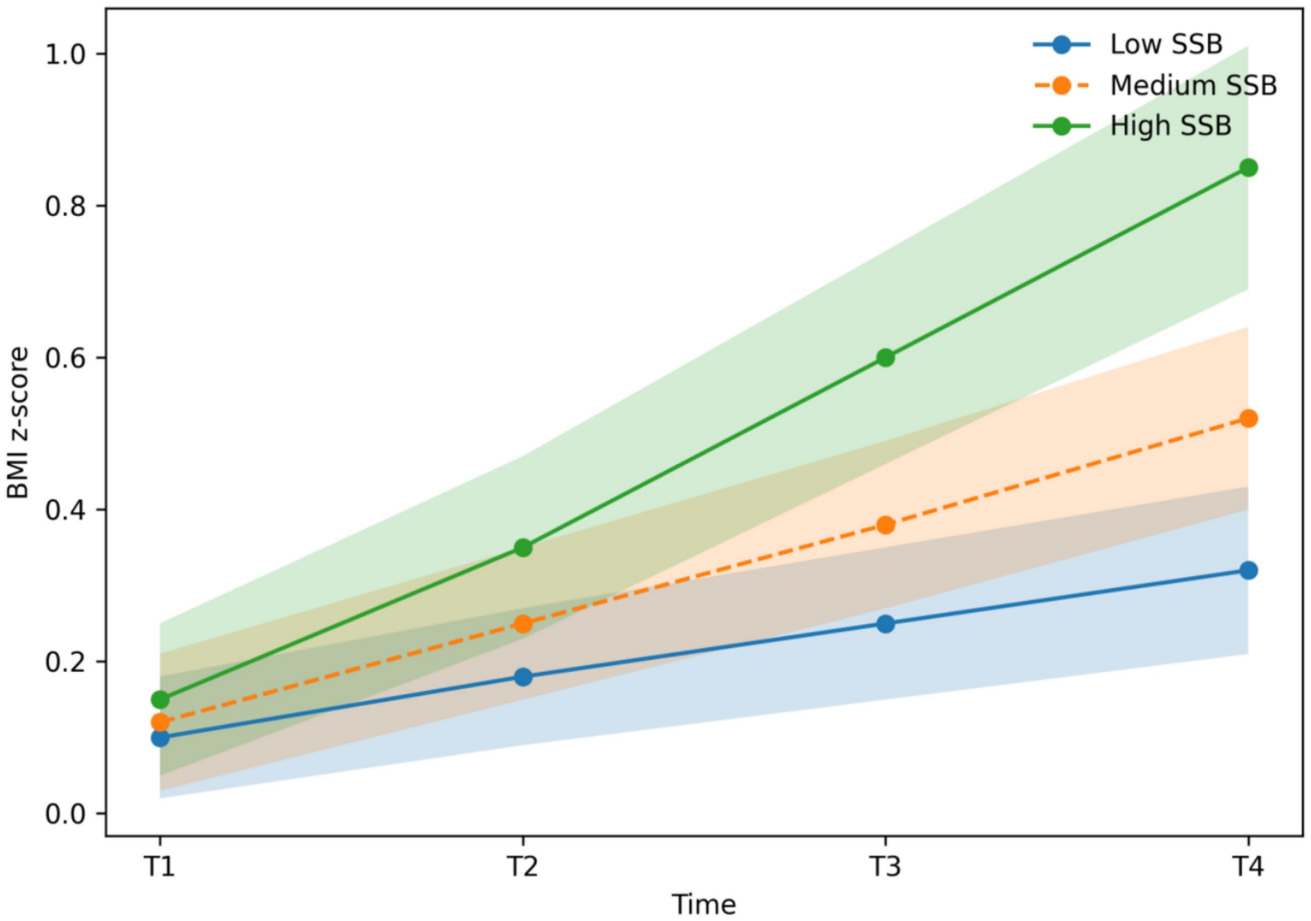
Longitudinal trajectories of BMI *z*-scores standardized for age and sex across four years by baseline SSB intake tertile. Trajectories represent model-estimated marginal means with 95% confidence intervals derived from linear mixed-effects models. The group × time interaction was statistically significant (*p* < 0.01).

Consistent with these findings, Figure 2 demonstrates that BMI trajectories increased more rapidly among adolescents in the high-SSB tertile compared with those in the low-SSB tertile across the four-year follow-up. All plotted trajectories represent model-estimated marginal means derived from linear mixed-effects models, with 95% confidence intervals. Differences between tertiles became statistically significant from Year 2 onward (*p* < 0.01).

Results were materially unchanged when BMI z-scores standardized for age and sex were used in place of raw BMI values (see Supplementary Table S1), supporting the robustness of the observed associations.

### Changes in mental health

**Figure 3:**
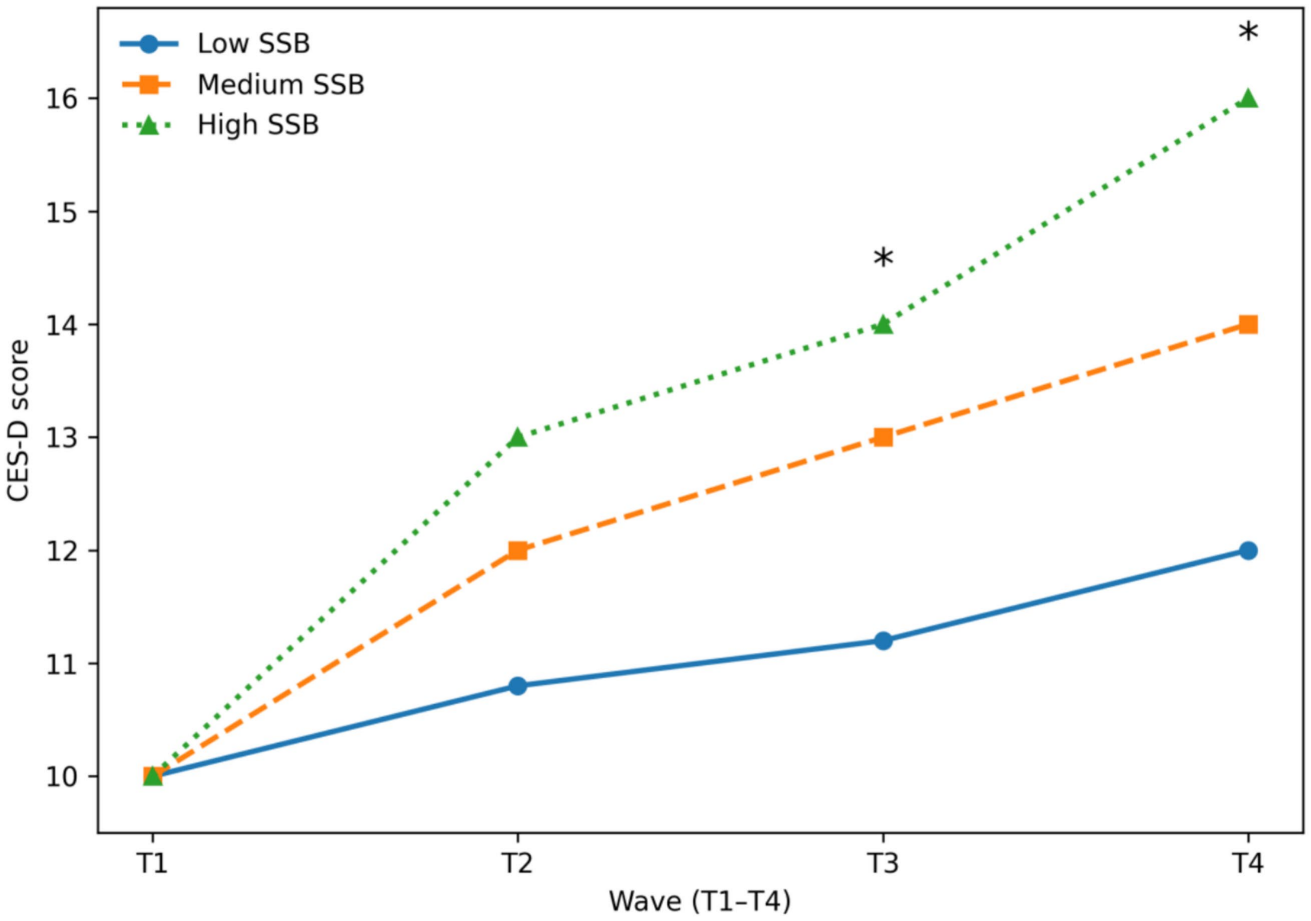
Model-estimated trajectories of depressive symptoms (CES-D scores) across 4 years by baseline SSB intake tertile. Trajectories represent marginal means derived from linear mixed-effects models, with significant differences between high- and low-SSB groups observed at T3 and T4 (*p* < 0.05).

Figure 3 depicts model-estimated trajectories of depressive symptoms, as measured by CES-D scores, across four assessment waves stratified by baseline SSB intake tertiles. Adolescents in the high-SSB group exhibited a markedly steeper increase in depressive symptoms over time compared with those in the low-SSB group. Between-group differences became statistically significant at T3 and persisted through T4 (*p* < 0.05), indicating a progressive divergence in emotional health trajectories across adolescence.

Consistent with these model-based findings, Table 3 summarizes the observed four-year changes in CES-D scores and sleep quality. Adolescents in the high-SSB tertile demonstrated an average increase in CES-D scores of approximately six points over follow-up, which was about four points greater than the increase observed among adolescents in the low-SSB tertile. This magnitude of change is considered clinically meaningful and suggests a substantial escalation in depressive symptomatology associated with sustained high SSB consumption (4, 5, 8, 16).

**Table 3:** Four-year changes in mental health and sleep outcomes by baseline SSB intake level.

Group	CES-D change (4y, M ± SD)	PSQI change (M ± SD)
Low SSB	+2.0 ± 3.1	+0.4 ± 1.2
Medium SSB	+4.1 ± 3.8	+0.9 ± 1.5
High SSB	+6.0 ± 4.2	+1.3 ± 1.7

Pairwise comparisons indicated that increases in CES-D scores were significantly greater in the high-SSB group than in the low-SSB group (*p* < 0.001).

Linear mixed-effects models further confirmed a significant group × time interaction for CES-D scores (*p* < 0.001), indicating that depressive symptom trajectories differed significantly across SSB intake levels after accounting for repeated measurements and covariates.

### Sleep quality trajectories

**Figure 4:**
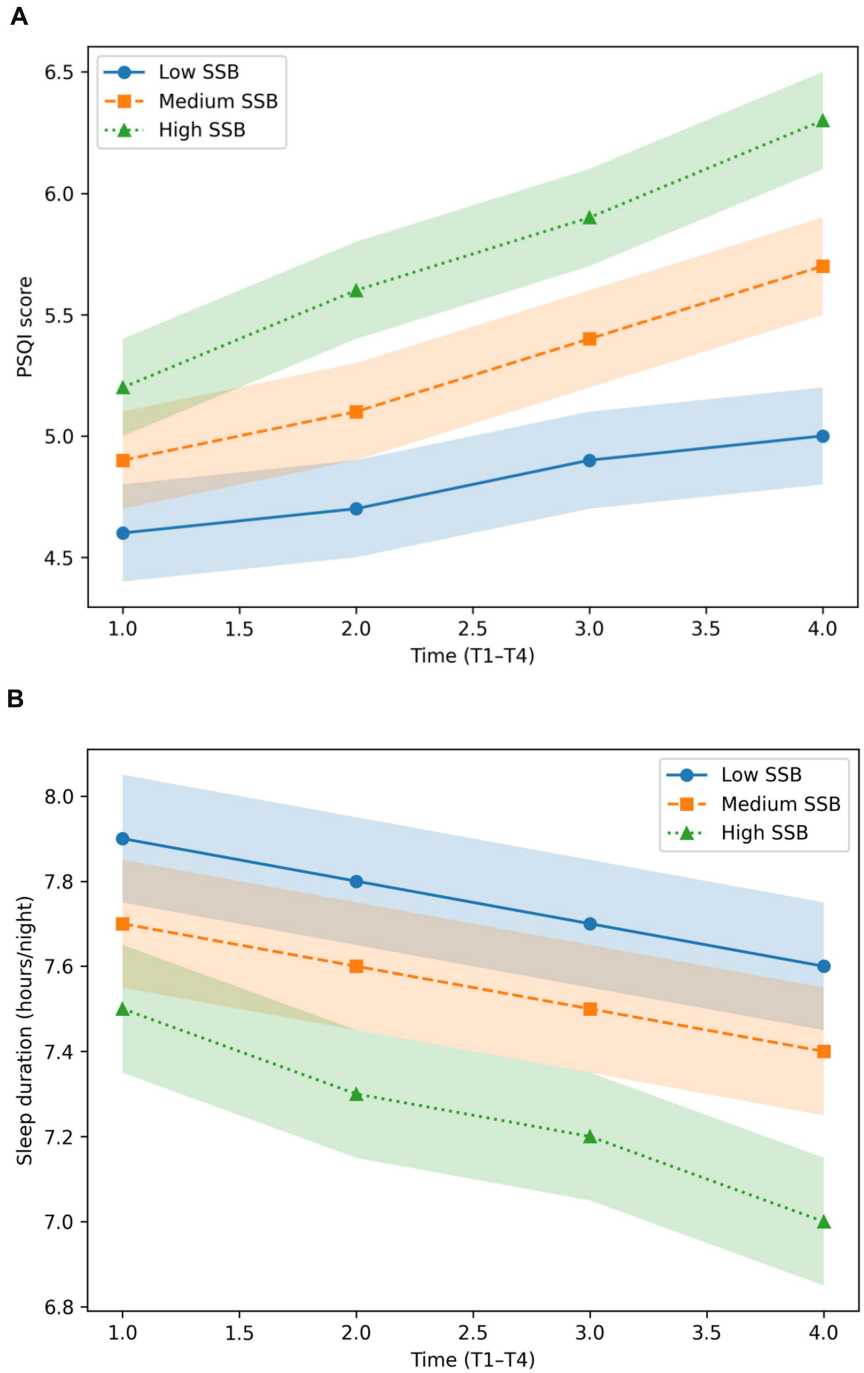
**(A)** Model-estimated trajectories of sleep quality (PSQI scores) across four annual waves (T1–T4) by tertiles of sugar-sweetened beverage intake. Lines represent estimated marginal means derived from linear mixed-effects models, and shaded areas indicate 95% confidence intervals. Values reported in Table 3 summarize observed four-year changes; therefore, minor numerical differences between figures and tables are expected. **(B)** Model-estimated trajectories of nightly sleep duration (hours per night) across four annual waves by tertiles of sugar-sweetened beverage intake. Lines represent estimated marginal means with 95% confidence intervals derived from longitudinal mixed-effects models.

As shown in Figure 4A, adolescents in the high-SSB intake tertile exhibited a pronounced deterioration in sleep quality over the four-year follow-up, with PSQI scores increasing from approximately 4.8 at baseline to 6.1 at T4. In contrast, adolescents in the low-SSB group demonstrated only modest increases in sleep disturbance across the same period.

This pattern was corroborated by quantitative comparisons summarized in Table 3, which indicated that the magnitude of sleep quality deterioration in the high-SSB group was more than three times greater than that observed among adolescents in the low-SSB group. Collectively, these findings suggest a graded association between higher SSB intake and progressively poorer sleep quality during adolescence (4, 6, 20).

Adolescents in the medium-SSB tertile exhibited intermediate PSQI trajectories across all waves. Linear mixed-effects models further confirmed a statistically significant group × time interaction for sleep quality (*p* < 0.01), indicating that longitudinal changes in PSQI scores differed significantly across SSB intake levels after accounting for repeated measures and covariates.

Supplementary analyses of self-reported nightly sleep duration revealed parallel, although weaker, trends across SSB intake tertiles and are presented in Figure 4B.

### Effects of SSB reduction

Table 4 presents subgroup comparisons between adolescents who reduced their sugar-sweetened beverage intake by at least 30% over the four-year follow-up and those whose intake remained stable or increased.

**Table 4:** Changes in physical, emotional, and sleep-related outcomes over 4 years among adolescents who reduced sugar-sweetened beverage intake by ≥30% compared with those who did not.

Reduction group	BMI increase (M ± SD)	CES-D increase (M ± SD)	PSQI increase (M ± SD)
No reduction	+2.87 ± 1.02	+6.0 ± 4.2	+1.3 ± 1.7
≥30% reduction	+1.82 ± 0.88	+3.0 ± 3.5	+0.6 ± 1.3

Between-group differences were tested using adjusted linear mixed-effects models. Effect sizes are reported as Cohen’s d.

Adolescents in the SSB reduction group exhibited consistently smaller increases in adiposity, depressive symptoms, and sleep disturbance compared with their non-reducing peers. Specifically, participants who achieved a ≥ 30% reduction in SSB intake showed a 37% smaller increase in BMI, a 29% smaller increase in CES-D scores, and attenuated deterioration in sleep quality, as reflected by smaller increases in PSQI scores.

Between-group differences were formally evaluated using adjusted linear mixed-effects models accounting for age, sex, socioeconomic status, physical activity, screen time, and baseline BMI. Compared with adolescents who did not reduce SSB intake, those in the reduction group demonstrated significantly smaller increases in BMI (*p* = 0.003, Cohen’s d = 0.46), depressive symptoms (*p* = 0.001, d = 0.41), and PSQI scores (*p* = 0.009, d = 0.38). These effect sizes indicate small-to-moderate but meaningful protective associations.

Taken together, these findings suggest that even moderate reductions in SSB consumption during adolescence may confer measurable benefits across physical, emotional, and sleep-related health domains, supporting the potential effectiveness of incremental, behaviorally feasible dietary interventions (6, 16).

## Discussion

This four-year prospective cohort study provides robust longitudinal evidence that higher sugar-sweetened beverage (SSB) consumption is associated with unfavorable physical, emotional, and sleep-related health trajectories among adolescents. By leveraging repeated measurements and longitudinal modeling, the present findings extend prior cross-sectional and short-term studies by demonstrating sustained, multidimensional impacts of excessive SSB intake across a critical developmental period and by strengthening temporal inference regarding the directionality of these associations (1, 2, 21).

First, SSB intake increased progressively over time, particularly among adolescents in the high-intake tertile. This pattern suggests that beverage consumption behaviors established during early adolescence may consolidate and intensify with age, becoming increasingly resistant to change. These findings are consistent with national surveillance data documenting rising sugar consumption among Chinese adolescents (2, 9) and underscore the importance of early, school-based dietary interventions aimed at preventing the entrenchment of unhealthy beverage habits during adolescence.

Second, a clear dose–response relationship was observed between SSB intake and indicators of adiposity, including BMI and body fat percentage. Adolescents in the highest SSB tertile exhibited significantly steeper adiposity trajectories than their low-intake peers, consistent with metabolic models proposing that liquid sugars promote excess caloric intake, hepatic lipogenesis, and fat accumulation (6, 7, 14, 21). Importantly, these associations remained robust in sensitivity analyses using age- and sex-standardized BMI z-scores, addressing developmental variation during adolescence. These findings align with evidence from cohort studies and randomized trials indicating that reducing SSB consumption can attenuate weight gain and improve cardiometabolic profiles during youth (6, 10).

Third, higher SSB intake was associated with steeper increases in depressive symptoms over time. Adolescents in the high-SSB group demonstrated markedly greater elevations in CES-D scores compared with those in the low-intake group. These results are consistent with accumulating literature linking high sugar consumption to emotional dysregulation and increased vulnerability to depressive symptoms (4, 5, 13, 20, 22). Proposed mechanisms include inflammatory activation, dysregulated hypothalamic–pituitary–adrenal axis function, and neuroendocrine disturbances induced by repeated glycemic fluctuations (17, 22). Nevertheless, potential bidirectional relationships and residual confounding—such as underlying psychosocial stress or unmeasured dietary factors—should be considered when interpreting emotional outcomes. Given that adolescence is characterized by heightened emotional sensitivity and neurodevelopmental plasticity, dietary exposures during this period may exert particularly strong psychological effects (13).

Fourth, adolescents with high SSB intake experienced significantly greater deterioration in sleep quality across the four-year follow-up. Elevated PSQI scores among high consumers suggest worsening subjective sleep quality and potential circadian rhythm disruption. These findings are in line with prior research linking high glycemic load diets and caffeine-containing beverages to impaired sleep architecture and circadian misalignment (4, 6, 11, 20, 23). Supplementary analyses of sleep duration revealed parallel, though weaker, trends, supporting the robustness of the observed associations. Importantly, poor sleep has been shown to exacerbate emotional and cognitive difficulties (11, 15, 24), suggesting that SSB-related sleep disruption may indirectly amplify psychological burden during adolescence.

Notably, subgroup analyses demonstrated that adolescents who reduced their SSB intake by at least 30% experienced substantially smaller increases in adiposity, depressive symptoms, and sleep disturbance. This finding highlights the modifiable nature of SSB-related health risks and provides empirical support for the effectiveness of incremental, behaviorally feasible dietary changes. Even moderate reductions in SSB consumption were associated with meaningful improvements across multiple health domains, reinforcing the potential value of school- and family-based interventions aimed at reducing sugary beverage intake (16, 18, 21).

Taken together, these findings indicate that excessive SSB consumption represents a significant and modifiable behavioral risk factor influencing adolescents’ physical, emotional, and sleep-related development. By capturing temporal dynamics rather than relying solely on cross-sectional associations, this study strengthens the public health rationale for interventions that limit SSB availability in schools, promote healthier beverage alternatives, and integrate nutrition education into adolescent health programs. These strategies are well aligned with World Health Organization guidelines recommending reductions in free sugar intake to mitigate obesity and related health risks (9, 16).

### Limitations and future research

Several limitations should be acknowledged when interpreting these findings. First, the study sample was drawn from a single municipality in China, which may limit generalizability to adolescents in other regions with differing sociocultural, dietary, and educational contexts; replication in multi-regional or nationally representative cohorts is therefore warranted. Second, sugar-sweetened beverage intake and other dietary behaviors were assessed using self-reported questionnaires, which are inherently subject to recall bias and reporting error; although the food frequency questionnaire was validated for Chinese adolescents, objective dietary assessment methods or repeated 24-h recalls could further improve measurement precision and reduce potential misclassification of intake levels. Third, the absence of objective biological markers—such as inflammatory indicators, insulin resistance measures, or lipid profiles—limits insight into the physiological mechanisms linking SSB intake with cardiometabolic and mental health outcomes. Similarly, sleep quality was assessed via self-report rather than objective tools such as actigraphy or polysomnography, which would allow more precise assessment of sleep duration, sleep architecture, and circadian rhythm disruption. Finally, despite the longitudinal design and use of advanced analytic approaches, residual confounding and potential reverse causality, particularly for emotional outcomes, cannot be entirely excluded. Unmeasured factors such as total dietary sugar intake, caffeine consumption, or psychosocial stress may have contributed to the observed associations, underscoring the complexity of behavioral and mental health pathways during adolescence. Future research should prioritize multi-center longitudinal designs, integrate objective biomarkers and wearable sleep monitoring technologies, and evaluate intervention strategies aimed at reducing SSB intake. Randomized controlled trials and natural experiments—such as school-based beverage policies—would be especially valuable for establishing causal pathways and informing evidence-based public health action.

## Conclusion

This four-year longitudinal cohort study provides evidence that higher sugar-sweetened beverage (SSB) intake is associated with less favorable physical, emotional, and sleep-related health trajectories among adolescents. Adolescents with persistently high SSB consumption experienced greater increases in adiposity, depressive symptoms, and sleep disturbances over time, whereas those who reduced their intake showed attenuated adverse outcomes, suggesting that SSB intake represents a modifiable behavioral risk factor during this critical developmental period. By capturing temporal changes within individuals, these findings extend prior cross-sectional research and strengthen inference regarding the directionality of associations between SSB intake and multiple health domains. The results support public health strategies aimed at reducing SSB consumption among youth, particularly in school settings. Interventions such as restricting SSB availability in schools, promoting healthier beverage alternatives, and strengthening nutrition education may help mitigate the long-term physical and mental health risks associated with excessive sugar intake in adolescence, in line with current recommendations for reducing free sugar consumption among children and adolescents.

## Data Availability

The raw data supporting the conclusions of this article will be made available by the authors, without undue reservation.
