# Supplementary material for: High sugar-sweetened beverage intake predicts adverse physical, emotional, and sleep health trajectories in adolescents: a 4-year prospective cohort study
Source: Front Public Health. 2026 Jan 14;13:1754072. doi: 10.3389/fpubh.2025.1754072 (PMC12847052; doi:10.3389/fpubh.2025.1754072)
Supplement: Supplementary file 1 [file Table_1.pdf]

Table S1. Four-year changes in BMI z-scores standardized for age and sex by baseline SSB intake tertile

**SSB intake tertile**	**BMI *z*-score change (4y, mean ± SD)**
Low SSB	+0.42 ± 0.31
Medium SSB	+0.61 ± 0.36
High SSB	+0.78 ± 0.41
