# Supplementary material for: High sugar-sweetened beverage intake predicts adverse physical, emotional, and sleep health trajectories in adolescents: a 4-year prospective cohort study
Source: Front Public Health. 2026 Jan 14;13:1754072. doi: 10.3389/fpubh.2025.1754072 (PMC12847052; doi:10.3389/fpubh.2025.1754072)
Supplement: Supplementary file 2 [file Image_1.pdf]

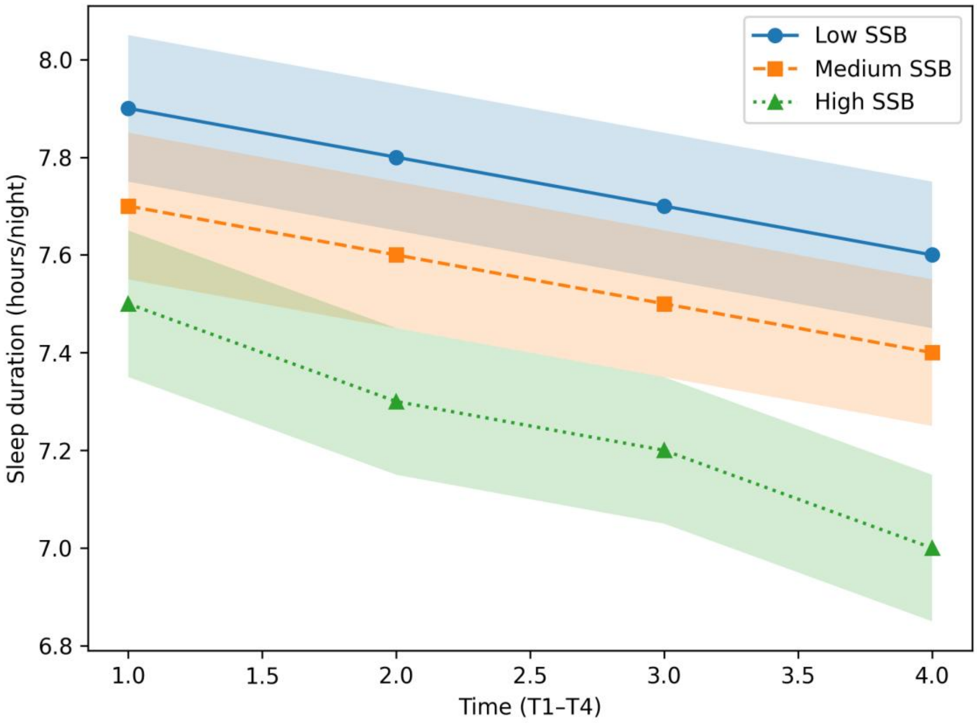

Figure 4B. Model-estimated trajectories of nightly sleep duration (hours per night) across four annual waves by tertiles of sugar-sweetened beverage intake. Lines represent estimated marginal means with 95% confidence intervals derived from longitudinal mixed-effects models.
